# An Up-to-Date Review Regarding the Biological Activity of *Geranium robertianum* L.

**DOI:** 10.3390/plants14060918

**Published:** 2025-03-14

**Authors:** Diana Haj Ali, Adriana Maria Dărăban, Diana Ungureanu, Adina Căta, Ioana Maria Carmen Ienașcu, Stefania Dinu, Cristina Adriana Dehelean, Corina Danciu

**Affiliations:** 1 Faculty of Pharmacy, Victor Babeș University of Medicine and Pharmacy Timișoara, Eftimie Murgu Square, No.2, 300041 Timișoara, Romania; diana.haj-ali@umft.ro (D.H.A.); cadehelean@umft.ro (C.A.D.); 2 Research Center for Pharmaco-Toxicological Evaluation, Victor Babeș University of Medicine and Pharmacy Timișoara, Eftimie Murgu Square, No.2, 300041 Timișoara, Romania; 3 Doctoral School, Victor Babeș University of Medicine and Pharmacy, Timișoara, Eftimie Murgu Square, No.2, 300041 Timișoara, Romania; diana.similie@umft.ro; 4 Faculty of Pharmacy, “Vasile Goldiș” Western University of Arad, 86 Liviu Rebreanu Street, 310045 Arad, Romania; nicole_adrianne10@yahoo.com; 5 Department of Pharmacognosy, Faculty of Pharmacy, Victor Babeș University of Medicine and Pharmacy Timișoara, Eftimie Murgu Square, No.2, 300041 Timișoara, Romania; corina.danciu@umft.ro; 6 Research and Processing Center of Medicinal and Aromatic Plants, Victor Babeș University of Medicine and Pharmacy Timișoara, Eftimie Murgu Square, No.2, 300041 Timișoara, Romania; 7 National Institute of Research and Development for Electrochemistry and Condensed Matter, 144 Dr. A. P. Podeanu, 300569 Timişoara, Romania; adina.cata@yahoo.com (A.C.); imcienascu@yahoo.com (I.M.C.I.); 8 Department of Pharmaceutical Sciences, Faculty of Pharmacy, “Vasile Goldiș” Western University of Arad, 86 Liviu Rebreanu, 310045 Arad, Romania; 9 Department of Pedodontics, Faculty of Dental Medicine, Victor Babeș University of Medicine and Pharmacy Timișoara 9 No., Revolutiei Bv., 300041 Timișoara, Romania; 10 Pediatric Dentistry Research Center, Faculty of Dental Medicine, Victor Babeș University of Medicine and Pharmacy Timișoara, 9 No., Revolutiei Bv., 300041 Timișoara, Romania

**Keywords:** taxonomy, phytochemical composition, antimicrobial, antiviral, anti-cancer, anti-inflammatory, antidiabetic, antioxidant, neuroprotective, antiulcerative, wound healing

## Abstract

In this modern era, in which interest in natural compounds is gaining more ground, *Geranium robertianum* L. (Gr), a species with long use in traditional medicine, stands out for its promising potential in managing a multitude of health issues. In this context, the present review aims to explore the main phytocompounds detected in various types of extracts, as well as the biological activity of Gr species. This review was conducted by analyzing data published up to February 2025 from peer-reviewed journals and databases including PubMed, Web of Science, and Google Scholar, using key words combinations such as *Geranium robertianum* L. and the searched phytocompound and biological effect. According to the literature the main phytochemical classes identified in different types of extracts include tannins, flavonoids, phenolic acids, and essential oils. The most important biological actions described in the literature are antioxidant, anti-inflammatory, antimicrobial, antiulcerative, neuroprotective, anti-cancer, and antidiabetic effects. However, knowledge about Gr is still relatively limited, requiring more detailed study regarding its pharmacological proprieties and the molecular mechanisms behind them.

## 1. Introduction

In recent decades, by fusing antique wisdom with advanced modern technologies, the exploration of plant-derived compounds has emerged as an evolving field. This quick development has facilitated a deeper understanding of the mechanisms by which these compounds interact with their biological targets [1].

Natural remedies are certainly among the earliest effective treatments used by humanity since ancient times [2]. Over the course of time, due to the inherent lack of diversity in synthetic molecules, which has ended up in decreasing the approval rate for new medicines, the interest in naturally derived drugs has increased [3]. Many of the molecules used today in treating certain diseases are of natural origin. Common examples can refer to aspirin, which was first extracted from *Salix alba* L., and the natural opioid morphine, extracted from *Papaver somniferum* L. Other examples can include digoxin from *Digitalis lanata* L., the antimalarial quinine extracted from *Cinchona officinalis* L., and the anti-cancer Paclitaxel extracted from *Taxus brevifolia* Nutt. [4,5,6]. The perpetual research about medicinal herbs has led to the identification of additional bioactive compounds with potential clinical applications and effectiveness. These ongoing studies highlighted the importance of nature as a provider of active molecules that keep contributing to the development of innovative drugs [7].

Nowadays, herbal therapy is widely recognized as an alternative and adjuvant treatment method for various acute and chronic diseases. Despite all its benefits, phytotherapy has its limitations, and in some cases it may be accompanied by side effects and interactions with other co-administrated natural or synthetic drugs [8]. One prominent limitation of phytotherapy is the lack of standardization in herbal drugs, which vary frequently in terms of active compounds and concentrations, in contrast to medicine based on synthetic drugs, where standardization is strictly defined. Another limitation is the low reproducibility of the findings of preclinical and clinical studies about natural-derived drugs, which remains one of the biggest challenges in phytotherapy. This unsatisfactory consistency and reproducibility may be caused mostly by the variations in active compounds between analyzed formulations dependent on the geographic region of cultivation, season, and extraction procedures. In order to reduce these limitations, specialists focus on a better standardization of plant extracts, implementation of efficient quality control systems, and conducting more rigorous clinical studies involving natural drugs in the form of standardized extracts [9,10]. Moreover, the current advancements in genomics, bioinformatics, and modern biotechnology have substantially boosted the rate of detection and in-depth study of phytocompounds, revealing their undeniable diversity [11].

## 2. Taxonomy

Due to its pharmacological properties, *Geranium robertianum* L. (Gr) has been included in traditional medicine across various cultures and remains an object of study, focused on expanding the knowledge of its therapeutic potential and its applications in modern herbal medicine [12]. Gr, commonly recognized as Herb Robert or Red Robin, is an herbaceous, annual or biannual species that mainly grows in shaded woodland environments, in addition to grassy areas and stony soils. Its native habitat extends across multiple continents, such as Europe, Asia, North America, and North Africa [13,14]. Taxonomically, Gr is classified in the kingdom *Plantae.* It is included in the *Tracheobionta* subkingdom and *Spermatophyta* superdivision, being part of the division *Magnoliophyta.* This plant belongs to the class *Magnoliopsida*, and it is recognized as a member of the *Rosidae* subclass. Regarding the order, Gr is placed in the *Geraniales*. This plant species belongs to the family *Geraniaceae*. The genus of this plant is *Geranium* L. and its species is *Geranium robertianum* L. [15]. There are identified three subspecies of Gr, each of which is associated with a specific geographic distribution. The first one is *Geranium robertianum* subsp. *robertianum*; its natural habitat has a wide range, allowing it to occur in countries with a temperate climate in the northern part of the Earth [16]. The second one is *Geranium robertianum* subsp. *maritimum*; it is mainly found in European countries, especially in the coastal regions of the United Kingdom, Germany, France, and Denmark [17]. The third subspecies is *Geranium robertianum* subsp. *celticum*; it is also originally from Europe [18]. The plant species is distinguished by its distinctive, irritating smell. It can grow between 10 and 60 cm high. The triangle-shaped leaves are compound, formed by multiple leaflets with lobed or serrated margins adhered to a single petiole. In terms of color, the leaves of *Geranium robertianum* L. usually have a pale green shade, which can turn into a slight red tint. The flowers are long-petiolate with five separated petals placed radially surrounding the ovary. The flowers are distinguished by their pinkish-purple color [19,20].

## 3. Phytochemical Composition

According to literature, polyphenols are the main active compounds of Gr, exhibiting quantitative variations dependent on the geographic region of cultivation and the specific type of analyzed extract [21]. The predominant phenolic constituents of Gr extracts include tannins, flavonoids, and phenolic acids such as ellagic acid, caffeic acid, gallic acid, and ferulic acid. Based on Hegnauer’s dictionary of plant chemistry, the first phytochemicals detected in Gr extracts were tannins, upon the identification of ellagic acid, which is an ellagitanin, a class of hydrolysable tannins that comprise hexahydroxydiphenic (HHDP) acid units esterified to a core polyol [19].

Paun et al. determined the polyphenolic content of Gr concentrated aqueous extract from whole plant cultivated in Romania using UV-Vis spectroscopy and HPLC-MS methods. The findings of this assay showed that the total phenolic compounds content expressed as gallic acid equivalents/L was 795.6  ±  8.2 (mg GAE/L), the total tannins content expressed as tannic acid equivalents/mL was 283.1  ±  5.4 (µg TA/mL), and the total flavonoid content expressed as quercetin equivalents/L was 168.1  ±  4.3 (mg QE/L). According to this study, the content of this extract in phenolic acids, such as ellagic acid and gallic acid, was 900.13 mg/kg and 1070.78 mg/kg, respectively [22]. Fodorea et al. identified, in hydrolyzed dried materials from whole plant of Gr, which was also cultivated in Romania, caffeic acid (6.62 µg/100 mg), caftaric acid (47.41 µg/100 mg), and ellagic acid (10,550.65 µg/100 mg). The non-hydrolyzed materials content in ellagic acid was 7599.76 µg/100 mg. These quantifications were performed using HPLC analysis [23]. The condensed tannins represented by proanthocyanidins were quantified by Ben Jemia et al., who determined proanthocyanidins in methanol extracts of plants from Tunisia; the results indicated a concentration of 0.86 mg catechin equivalents/g dry weight [19,24].

The ellagitannin geraniin was isolated for the first time from the species *Geranium thunbergii* Sieb. et Zucc.; later, it was established that geraniin is the principal active compound of species belonging to the *Geraniaceae* and *Euphorbiaceae* families. After that, Okuda et al. purified geraniin from an acetone/water extract of leaves collected in Japan from *G. thunbergii*, finding that geraniin represents approximately 10% of the dried leaf’s weight [25]. Geraniin was also detected in aqueous extracts of plants originally from Poland by Piwowarski et al. [26].

A study conducted by Graça et al. evaluated the phytochemical compositions of whole plant ethyl acetate, methanol, and acetone extracts of Gr. The extracts were fractionated by gradient elution column chromatography on silica gel. To quantify the polyphenolic content, a HPLC-DAD-ESI/MS was performed. The results revealed the presence of 17 flavonoid glycoside derivatives, represented primarily by quercetin and kaempferol derivatives and naringenin-7-O-glucoside. The most concentrated heteroside of kaempferol (kaempferol-*O*-deoxyhexosyl-glucuronide) was detected in the methanol extract at a concentration of 4.78 ± 0.11 mg/g, followed by kaempferol-*O*-deoxyhexosyl-hexoside at 2.61 ± 0.07 mg/g in the acetone extract and kaempferol-*O*-deoxyhexosyl-glucuronid at 2.32 ± 0.05 mg/g in the ethyl acetate extract. Regarding quercetin heterosides, the highest concentration was scored for quercetin-3-*O*-rutinoside at 3.39 ± 0.06 mg/g in the acetone extract, followed by quercetin-*O*-deoxyhexosyl-hexoside at 1.71 ± 0.02 mg/g in the methanol extract and quercetin-*O*-deoxyhexosyl-glucuronide at 0.9421 ± 0.0004 mg/g in the ethyl acetate extract [27].

The first characterization of the content in essential oils of Gr species was carried out by Pedro et al. in 1992, using the aerial part of plants cultivated in the Netherlands, where higher levels of essential oils compounds were reported for linalool (22.9%), γ-terpinene (13.9%), germacrene D (7.8%), limonene (5.3%), and geraniol (4.4%) [28]. Later, in 2012, Radulovic et al. isolated essential oils from the aerial and underground parts of Gr cultivated in Serbia by hydro-distillation. The quantitative detection was performed using GC-MS. The findings of this analysis indicate the identification of 152 components for the aerial part and 53 for the underground part. The most prevalent compounds were hexadecanoic acid (45.3%) and pentacosane (28.5%) in the underground part and hexadecanoic acid (16.6%), hexahydrofarnesyl acetone (6.5%), and caryophyllene oxide (5.4%) in the aerial part [29]. The main phytocompounds and essential oil constituents of various types of Gr extracts are mentioned in Table 1.

The nutrient analysis conducted by Neagu et al. at 8%, 10%, and 15% (mass concentration) hydroalcoholic extracts in 50%, 70%, and 96% alcohol, prepared from leaves of Gr cultivated in Romania, revealed the presence of proteins in concentrations ranging between 1.262 and 2.40 µg/mL and sugar in concentrations ranging between 153.10 and 661.60 µg/mL. The greatest levels of proteins and sugars were observed in 10% (mass concentration) extract in 70% ethanol, while the lowest levels were found in 15% (mass concentration) in 96% ethanol [30].

Regarding the vitamin content of Gr species, Igwenyi et al. analyzed the macerated fresh leaves’ content of vitamins. The leaves of Gr were collected from plants from Abakaliki, Nigeria. The results of this analysis indicated that Gr is a rich source of hydrosoluble vitamins such as vitamins B complex (vitamin B2 being the most concentrated 818.21 ± 0.07 mg/100 g) and vitamin C (14.76 ± 5.1 mg/100 g), as well as in liposoluble vitamins A (1.44 ± 0.02 mg/100 g) and E (0.016 ± 0.02 mg/100 g). Igwenyi I et al. also determined the saponin content (1.43 ± 0.06 mg/100 g) and the alkaloid amount (1.20 ± 0.10 mg/100 g) [31].

**Figure 1:**
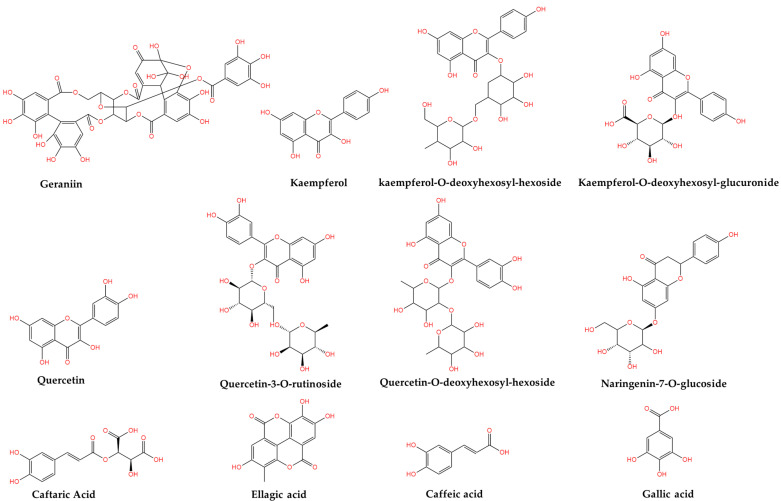
Chemical structures of the main phytocompounds of *Geranium robertianum* L. The figure was created using KingDraw V.3.6.1.

**Figure 2:**
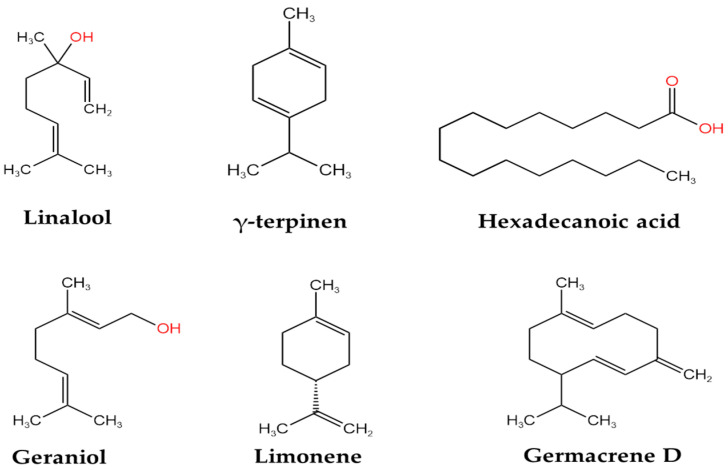
Chemical structures of the main terpenes present in the essential oils of *Geranium robertianum* L. The figure was created using KingDraw V.3.6.1.

In Figure 1 and Figure 2, the chemical structures of the main bioactive compounds of *Geranium robertianum* L. are represented.

Aqueous and hydroalcoholic extracts obtained from dried roots and leaves of Gr plants native to Romania were evaluated by Paun et al. in order to determine the concentrations of Ca, Mg, Mn, Zn, and Fe. These metals were detected in the extracts using a flame atomic absorption spectrometer (FAAS) in the following concentrations dependent on the extract type [19,32] (Table 2).

## 4. Biological Activity

Gr has been used since ancient times in folk medicine due to its multiple therapeutic properties. Different type of extracts obtained from this species are reported for their antimicrobial, antiviral, antioxidant, anti-inflammatory, anticancer, neuroprotective, hepatoprotective, hemostatic, antidiarrheal, antiallergic, antidiabetic, diuretic, stomachic, antiulcer, and immunomodulatory effects [14,19,31,33,34,35]. These therapeutic properties make it effective when administered internally, in various pharmaceutical forms or extracts, in the management of multiple pathologies, such as diarrhea, gastritis, ulcers, flu, sinusitis, diabetes, hypertension, hypercholesterolemia, cancer, rheumatism genitourinary problems, hemorrhages, and liver disorders [21,36,37,38,39]. In Deliblato Sands, Serbia, this plant is known and used for its antidiarrheal and antihemorrhagic (internally) and anti-inflammatory (externally) effects [36]. Furthermore, flowers or leaves infusion obtained from Gr were used for infertility problems in the South Tyrol area of northern Italy [37]. In the Finnish Flora Fennica, Gr is mentioned as a traditional use in the form of an infusion for the effect of expelling breast milk in postpartum women [39]. Topical administration of various extracts as such or after incorporation in different bases of herb Robert has been shown to be effective in treating wounds, burns, cold sores, varicose veins, scalp parasitosis, oropharyngeal inflammations (aphthous stomatitis, herpetic angina), and skin inflammations (including mosquito bites and mild rashes) [8,12,21,38,39,40]. The main therapeutic actions of Gr will be described below.

### 4.1. Antimicrobial Activity

The antimicrobial activity of herb Robert was first investigated in 2005 by Hersch-Martínez et al. using a commercially available volatile oil as a test. The test results of the Kirby–Bauer agar diffusion method did not confirm that the volatile oil was effective against pathogenic bacterial strains isolated from pediatric patients [19]. Subsequently, several studies appeared that attested the antimicrobial potential of Gr extracts.

Osiane Alhage et al. aimed to evaluate the antimicrobial potential of three Gr extracts (methanol, dichloromethane, and crude aqueous) produced from various parts of the plant by the plate-hole diffusion method. It was shown that the leaves dichloromethane extract significantly inhibited the growth of *S. aureus* (Gram-positive bacteria), showing an inhibition diameter of 8 mm at a concentration of 1 mg/mL. Furthermore, the only active extract against *Candida albicans* was the stems methanol extract, causing an inhibition diameter of 9 mm at a concentration of 10 mg/mL. This study also demonstrated that various extracts of Robert herb showed weak activity against *P. aeruginosa* and *E. coli* (Gram-negative bacteria) [33]. Another study conducted by Świątek et al. evaluated four extracts (methanolic, ethanolic, hexane, and aqueous) from the aerial parts of Gr against several bacterial and fungal strains. It was observed that, among the bacteria, only the Gram-positive ones were susceptible to the action of the extracts, the hexane one showing the strongest activity, with a MIC = 0.06–0.5 mg/mL against all of the species. The ethanolic extract was also active against Gram-positive bacteria, except for strains of MRSA (methicillin-resistant *Staphylococcus aureus*). Regarding antifungal activity, the methanolic and ethanolic extracts proved to be effective against the majority of the tested strains, with four species of *Candida* being among the most sensitive to their activity (*C. glabrata*, *C. tropicalis*, *C. krusei*, and *C. parapsilosis*), with a MIC = 1 mg/mL [41].

A hydroalcoholic extract (20:80) of herb Robert (whole plant) was shown to be efficient against several bacterial strains (*S. aureus*, *B. cerreus*, *E. coli*, *Campylobacter coli*, *Salmonella infantis*, and *Listeria monocytogenes*). Following the application of broth microdilution methods, the most sensitive species proved to be *S. aureus* (MIC = 2.77 mg/mL), while *B. cereus* and *L. monocytogenes* were less susceptible to the antibacterial action of the extract (MIC = 6.66 mg/mL) [42]. Additionally, the aqueous extract of the entire plant showed effectiveness against certain streptococcal species (*S. mutans* and *S. sobrinus*) associated with bacteria that cause dental caries [41].

Regarding the antibacterial properties of the volatile oils, Renda et al. demonstrated that the volatile oil obtained from *Geranii herba* exhibited similar antimicrobial activity against *S. aureus* ATCC 25923, *B. cerreus* 709 Roma, and *Mycobacterium smegmatis* ATCC 43251 (MIC = 0.805 mg/mL). However, it was ineffective against the *B. subtilis*, *Clostridium*, or streptococci species tested [43,44]. In the same line, Gębarowska et al. evaluated the antibacterial activity of essential oil extracted from the aerial parts of G. robertianum. The obtained results showed that certain Gram-positive (*S. aureus* displayed the lowest MIC = 1.25 mg/mL) and Gram-negative (*E. coli*) bacteria are sensitive to the activity of the volatile oil (in concentrations of 1.25 to 10 mg/mL). The inhibition of bacterial growth was attributed to the high content of monoterpenoids with -OH groups (geraniol, linalool), monoterpenes (γ-terpinene), and sesquiterpenes (β-caryophyllene), which represent over 30% of the volatile oil ingredients [45]. A clinical study demonstrated that the use of ear drops containing Robert’s herb, cloves, and lavender essential oils in human subjects with acute external otitis showed similar efficacy to the use of ear drops containing ciprofloxacin 0.3%, a fluoroquinolone antibiotic [46,47].

### 4.2. Antiviral

The antiviral activity of Gr can be attributed to the volatile oils, tannins, and geraniin (a bitter compound, the main component of *Geranii herba*) [48]. Several studies have stated that geraniin is effective against influenza viruses (Influenza A and B), herpes simplex virus, human immunodeficiency virus 1 (HIV-1), and dengue virus 2 (DENV2) [48]. Another polyphenolic compound with potent antiviral effect from the composition of Gr is ellagic acid, known for its effect against Zika virus, HIV-1, hepatitis B virus, Ebola virus, and some influenza viruses and rhinoviruses [48,49,50,51,52]. Additionally, the polyphenolic compounds found in the aerial parts of herb Robert may be beneficial in preventing or treating SARS-CoV-2 infection [48].

### 4.3. Anti-Inflammatory

The anti-inflammatory activity of various extracts of herb Robert have been demonstrated and analyzed in various studies. One of them refers to the ability to neutralize hypochlorous acid (the main oxidizing agent produced by neutrophils, which plays an important role in the inflammation process) of a commercially available 50% hydroalcoholic extract. It was observed that this extract moderately inhibited the oxidation of 5-thio-2-nitrobenzoic acid mediated by HOCl (IC50 = 111.94 ± 1.79 µM), having a weaker antioxidant and anti-inflammatory effect than quercetin, the positive control (IC50 = 34.22 ± 0.72 µM) [19]. Another study conducted by Piwowarski et al. analyzed the activity of an aqueous extract of *Geranii herba* against hyaluronidase and elastase, two enzymes involved in the degradation of the extracellular matrix, a process involved in the development of inflammatory diseases. At a concentration of 10 µg/mL, this extract inhibited hyaluronidase activity by 7.2% and elastase by 34.7%, suggesting a possible anti-inflammatory effect [53]. On the other hand, Catarino et al. showed that the leaves and stems of an aqueous extracts of Gr did not inhibit the activity of 5-LOX, the enzyme that catalyzes the oxidation of arachidonic acid to leukotrienes, up to a concentration of 60 µg/mL and did not modulate the expression of the inducible NO synthase [35].

### 4.4. Antioxidant

Antioxidants are necessary compounds in the body that neutralize reactive oxygen species (ROS), such as hydroxyl, hydrogen peroxide, and superoxide, preventing lipid peroxidation and DNA damage [27]. An excess of ROS is implicated in the occurrence of serious health problems including cancer, neurological disorders, heart disease, gastroduodenal ulcers, etc. The antioxidant activity of Gr extracts is closely correlated with the number of -OH groups available in the structure of the chemical compounds (mostly polyphenols) found in the respective extracts. Thus, it is known that the use of water and methanol as extraction solvents leads to the obtaining of extracts rich in flavonoids, compounds with strong antioxidant capacity [33]. The Gr polyphenolic content includes compounds such as ellagic acid, gallic acid, and caffeic acid [33]. A methanolic extract obtained from the roots of herb Robert exerted a marked antioxidant capacity, inhibiting 97% of the DPPH (2,2-diphenyl-2-picrylhydrazyl) radical at a concentration of 0.3 mg/mL [33]. Another study revealed that the methanol extract of Geranii folium demonstrated an antioxidant capacity (IC50 = 6.8 µg/mL) superior to butylhydroxytoluene, a food additive with an antioxidant role (IC50 = 85 µg/mL), in the beta-carotene/linoleic acid bleaching assay [24].

The aqueous extract of herb Robert leaves at concentration 400 mg/Kg obtained by Bawish et al. demonstrated a better radical scavenging capacity than aloe vera gel powder, with a value of 78.29 ± 4.59% within the DPPH test [14]. Another study showed that the infusion of the whole plant presented an EC50 of 65 ± 1 µg/mL in the DPPH assay. The antioxidant activity could be attributed to the significant polyphenol content expressed as gallic acid equivalents (228 ± 5 mg GAE/g) [21]. It was observed by Catarino et al. that infusions of leaves and stems of herb Robert possess good antioxidant properties. Following the application of DPPH and ABTS (2,2′-azino-bis(3-ethylbenzothiazoline-6-sulfonic acid)) assays, the leaf extract presented IC50 values of 7.6 µg/mL and 3.9 µg/mL, respectively, proving to have a more potent antioxidant activity than the stem extract, which had IC50 values of 17.3 µg/mL and 5.8 µg/mL. The IC50 values of the two extracts are inversely proportional to their total polyphenol content (649.2 mg/g for the leaves extract and 536.4 mg/g for the stems extract), which underlines that the antioxidant activity increases with increasing polyphenol content [35]. Neagu et al. also highlighted the correlation between the polyphenol content and antioxidant activity of Gr extracts. Moreover, they stated that concentrated extracts of herb Robert with very high antioxidant activity (over 92% DPPH inhibition) can be obtained through membrane processes to concentrate the polyphenols [34]. The antioxidant potential of methanolic extract (drug/solvent ratio 1:10) from the aerial and underground parts of Gr was evaluated by Ilić et al. by performing a FRAP (Ferric Reducing Antioxidant Power) and DPPH assays. The results of this analysis indicated a FRAP value of 6.33 ± 0.02 mmol Fe^2+^/g, and the scavenging of 50% of DPPH radicals (SC50) was 5.34 ± 0.11 µg/mL [54].

The antioxidant effect of Gr extracts may also be useful in preventing photoaging. Extracts from several medicinal plants, including herb Robert, were used in a cosmetic product that participated in a clinical trial to assess the improvement of periorbital wrinkles. However, the results did not show a significant improvement in the skin around the patients’ eyes following application of the product [55].

### 4.5. Anti-Cancer

Even though it has been traditionally used in the treatment of cancer in various territories, there are not many studies evaluating the anticancer activity of Gr. For example, several aqueous and organic extracts (acetone, ethyl acetate, methyl chloride, and n-hexane) showed cytotoxic potential (with IC50 values ranging from 55.68 to 236 µg/mL) against cancer cell lines such as HeLa (cervical adenocarcinoma), HepG2 (hepatocellular carcinoma), MCF-7(mammary gland adenocarcinoma), and NCI-H460 (lung carcinoma). Of these extracts, the most potent was the acetone extract, with IC50 values of 57–60 µg/mL [12].

Paun et al. evaluated the cytotoxic potential of two extracts concentrated by micro- and ultrafiltration from Gr leaves (one aqueous and one hydroethanolic 50:50) against the malignant Hep-2p cell line. The results of this study showed that the extracts possessed moderate cytotoxic potential against cancer cells and a very low toxicity against healthy cells. Of the two extracts, the most effective against malignant cells was proven to be the hydroethanolic one, an effect that can be attributed to the contained polyphenolic compounds. In the same line, Neagu et al. evaluated the anticancer potential of several purified and concentrated herb Robert aqueous extracts against the malignant human cell line Hep-2p. The results of the MTT (3-(4,5-dimethylthiazol-2-yl)-2,5 diphenyl tetrazolium bromide) assay showed that cell viability decreased with increasing dose and with increasing incubation period. Thus, purified and concentrated extracts from herb Robert can be considered candidates for cancer treatment [56]. It was also observed that bio-guided fractionation of some herb Robert extracts resulted in obtaining fractions with improved cytotoxic potential compared to the initial crude extracts [43].

Another study analyzed the antitumor activity of two aqueous extracts (infusion and decoction) and five organic extracts (methanol, dichloromethane, acetone, ethyl acetate, and n-hexane) of *G. robertianum* against four malignant human cell lines (MCF-7, NCI-H460, HeLa, and HepG2). All extracts showed cytotoxic activity. The acetone extract was the most active against MCF-7 (GI50 = 60 µg/mL) and HeLa (GI50 = 57 µg/mL) lines and the dichloromethane extract against NCI-H460 (GI50 = 66 µg/mL), while the herb Robert infusion showed the lowest GI50 against HepG2 (45.68 µg/mL). At the same time, the acetone extract showed the highest toxicity (GI50 approx. 176 µg/mL) towards PLP2 (normal primary porcine liver cells), used for hepatoxicity assessment, while the aqueous, h-hexane, and dichloromethane extracts did not show toxicity towards PLP2 at the tested concentrations [21].

Another series of experiments conducted by Swiatek determined the cytotoxicity of four extracts of *Geranii herba* (aqueous, methanolic, ethyl acetate, and hexane) on four cell lines (VERO—noncancerous; FaDu and Detroit 562—pharyngeal cancer; RKO—colon cancer). According to the MTT assay (72 h of incubation), the ethyl acetate extract was found to be the most cytotoxic against the FaDu (CC50 = 63.36 µg/mL) and RKO (CC50 = 63.63 µg/mL) cell lines, while the hexane extract was the most effective against the Detroit562 cell line (CC50 = 117.57 µg/mL). The methanol extract demonstrated the highest toxicity on VERO (CC50 = 187.17 µg/mL) [41]. In terms of selective toxicity, Catarino et al. showed that the infusion of Gr leaves does not show cytotoxic effect on HepG-2 (healthy liver cell line) at any of the tested concentrations (25–100 µg/mL, MTT test) [35].

The anticancer effect of Gr could be attributed to some phenolic acids that are part of the phytochemical composition of Geranium spp. These are gallic and ellagic acid, compounds with documented inhibitory action on carcinogenesis. Also, other compounds with a certified anticancer effect are geraniin, which is known to cause increased ROS accumulation and to trigger autophagy-mediated cell death of C666-1 (nasopharyngeal cancer cell line), and flavonoids (quercetin, kaempferol) that interfere with certain signaling pathways (MAPK, NF-κB, and PI3K/Akt) involved in the cancer process [41,57,58].

### 4.6. Wound-Healing

The certified antibacterial effect of herb Robert extracts may justify the empirical use of this plant in the treatment of difficult-to-heal wounds [41]. In the Balkan peninsula, more precisely in Montenegro, the aerial parts of this plant were traditionally used as an infusion for the treatment of poorly healing wounds [59].

### 4.7. Neuroprotective

Even if the antioxidant and anti-inflammatory properties of herb Robert have been known since ancient times, its neuroprotective potential has not been studied enough. Arslan and his collaborators investigated, for the first time, the neuroprotective effect of an aqueous extract from Gr leaves on an in vitro Parkinson’s disease model induced by MPP+(1-methyl-4-phenylpyridinium). The data obtained showed that the extract exhibited neuroprotective effects due to the modulation of antiapoptotic systems and the reduction of oxidative stress. Furthermore, it was observed that this extract modified the activity of the enzyme acetylcholinesterase. In conclusion, these results suggest that Gr extracts could be useful in the management of Parkinson’s disease [60].

### 4.8. Antiulcerative

The traditional use of decoctions or infusions of Gr in the treatment of digestive pathologies such as gastritis and ulcers is well known [21,45]. In traditional Peruvian medicine, the aqueous extract and decoction of herb Robert bark were used in the treatment of gastritis [33]. The study conducted by Bawish et al. aimed to evaluate the antiulcerative effect of Gr leaves and aloe vera gel powder on mice with acetylsalicylic acid-induced gastric ulcer. The data obtained showed that both presented a gastroprotective effect, also improving the anxiety states associated with the disease. Another beneficial effect of pretreatment with Gr leaves and aloe vera gel powder was the restoration of stomach architecture due to the reduction in inflammatory stress, TNF-α expression at the mucosal level, and oxidative stress-related genes (NF-KB, HO-1, and Nrf-2) due to the high polyphenol content [14].

### 4.9. Antidiabetic

**Figure 3:**
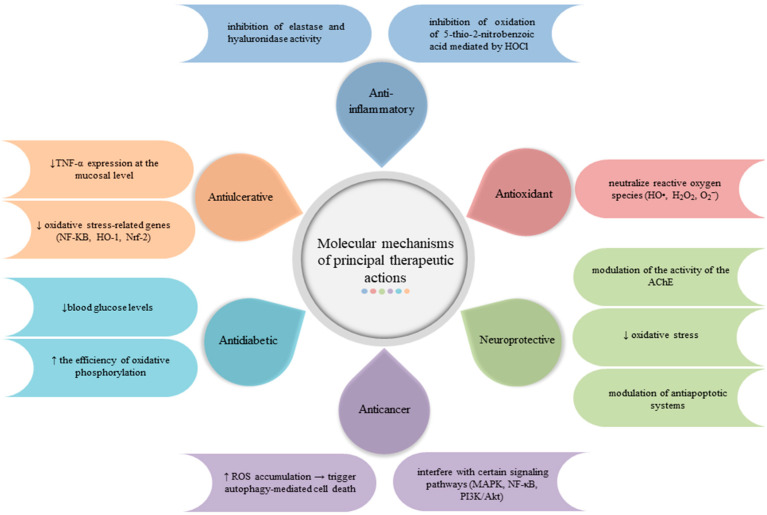
Molecular mechanisms of principal therapeutic action of *Geranium robertianum* L. extracts. (↑: increase, ↓: decrease). The figure was created using BioRender.com.

This plant was commonly used for its anti-hyperglycaemiant effect in Portuguese herbal medicine [61]. In traditional Algerian medicine, infusions of Gr leaves and flowers were known and used for their antidiabetic properties [62]. In order to assess this therapeutic action, Ferreira et al. orally administered *G. robertianum* leaf decoctions to mice with type 2 diabetes (Goto-Kakizaki model). The results obtained demonstrated a decrease in blood glucose levels, an improvement in liver mitochondrial respiratory parameters, and an increase in the efficiency of oxidative phosphorylation. Thus, herb Robert could be useful in improving mitochondrial function in patients with type 2 diabetes [60]. The molecular mechanism of the main therapeutic actions of Gr are highlighted and briefly described in Figure 3.

## 5. Conclusions

Evidence from the literature demonstrates that Gr, through its bioactive compounds such as flavonoids, tannin, essential oils, and phenolic acids, exerts antimicrobial, anticancer, antioxidant, anti-inflammatory, antidiabetic, neuroprotective, and antiulcerative effects and promotes wound healing. This highlights the potential relevance of this species in the therapy and prevention of various diseases. Despite all these encouraging data, knowledge about Gr remains relatively limited, requiring more in-depth study regarding its pharmacological activity and the mechanism of action.

## Figures and Tables

**Table 1:** Main phytocompounds and essential oil constituents of *Geranium robertianum* L.

Study Team and Year	Country	Compound	Concentration	Type of Extract/Plant Material	Method of Analysis
Okuda et al., 1980 [25]	Japan	Geraniin	10% of leaves dried weight	acetone extract	HPLC-UV
Paun et al., 2012 [22]	Romania	gallic acid ellagic acid	1070.78 mg/kg 900.13 mg/kg	aqueous extract	HPLC-MS
Fodorea et al., 2005 [23]	Romania	ellagic acid	7599.76 µg/100 mg	alcoholic extract (non-hydrolyzed materials)	HPLC-UV
		ellagic acid	10,550.65 µg/100 mg	alcoholic extract (hydrolyzed materials)	
		caffeic acid	6.62 µg/100 mg		
		caftaric acid	47.41 µg/100 mg		
Graça et al., 2017 [27]	Portugal	kaempferol-*O*-deoxyhexosyl-glucuronide	4.78 ± 0.11 mg/g	methanol extract	HPLC-DAD- ESI/MS
		kaempferol-*O*-deoxyhexosyl-hexoside	2.61 ± 0.07 mg/g	acetone extract	
		kaempferol-*O*-deoxyhexosyl-glucuronid	2.32 ± 0.05 mg/g	ethyl acetate extract	
		quercetin-3-*O*-rutinoside	3.39 ± 0.06 mg/g	acetone extract	
		quercetin-*O*-deoxyhexosyl-hexoside	1.71 ± 0.02 mg/g	methanol extract	
		quercetin-*O*-deoxyhexosyl-glucuronide	0.9421 ± 0.0004 mg/g	ethyl acetate extract	
Radulovic’ et al., 2012 [29]	Serbia	hexadecanoic acid	45.3%	diethyl ether extract from the underground part diethyl ether extract from the aerial part	GC-MS
		Pentacosane	28.5%		
		hexadecanoic acid	16.6%		
		hexahydrofarnesyl acetone	6.5%		
		caryophyllene oxide	5.4%		

**Table 2:** The content of *Geranium robertianum* L. aqueous and hydroalcoholic extracts in, Mg, Mn, Fe, Ca, and Zn.

Extract Type	Mg (mg/L)	Mn (mg/L)	Fe (mg/L)	Ca (mg/L)	Zn (mg/L)
Aqueous extract	10.40 ± 0.3	0.893 ± 0.07	3.2 ± 0.1	0.935 ± 0.08	0.071 ± 0.006
Hydro-alcoholic (50/50) extract	9.78 ± 0.7	0.819 ± 0.07	1.8 ± 0.1	0.927 ± 0.08	0.069 ± 0.006

## Data Availability

Data are contained within the article.

## Abbreviations

MIC	Minimum inhibitory concentration
NO synthase	Nitric oxide synthase
IC50	Half maximal inhibitory concentration
GI50	50% growth inhibition
CC50	Concentration of cytotoxicity 50%
LOX	Lipoxygenase
DNA	Deoxyribonucleic acid
EC50	Half maximal effective concentration
MAPK	Mitogen activated protein kinase
NF-κB	Nuclear factor Kappa B
PI3K/Akt	Phosphatidylinositol 3-kinase (PI3K)/protein kinase B (AKT)
TNF-α	Tumor necrosis factor
HO-1	Heme oxygenase
Nrf-2	Nuclear factor erythroid 2-related factor 2
